# The Mismatch Repair System (MMR) in Head and Neck Carcinogenesis and Its Role in Modulating the Response to Immunotherapy: A Critical Review

**DOI:** 10.3390/cancers12103006

**Published:** 2020-10-16

**Authors:** Maria Cilona, Luca Giovanni Locatello, Luca Novelli, Oreste Gallo

**Affiliations:** 1 Department of Otorhinolaryngology, Careggi University Hospital, Largo Brambilla, 3-50134 Florence, Italy; maria.cilona@unifi.it (M.C.); locatello.lucagiovanni@gmail.com (L.G.L.); 2 Department of Pathology, Careggi University Hospital, Largo Brambilla, 3-50134 Florence, Italy; novellil@aou-careggi.toscana.it

**Keywords:** microsatellite instability, MSI, MMR proteins, head and neck cancer, progression, local recurrence, multiple primary tumors, immunotherapy

## Abstract

**Simple Summary:**

The dysfunction of the mismatch repair system, an important mechanism for the detection and correction of DNA replication mistakes, may often lead to instability in the length of specific genetic sequences, known as microsatellites, and to the accumulation of mutations. Microsatellite instability is a well-known risk factor for the development of colorectal cancers and other types of tumors but is also considered a positive predictor of the immunotherapy response. Malignancies harboring such a specific genomic instability are very immunogenic because of the great number of aberrant antigens they produce. Therapies based on the blockade of specific immune checkpoints have shown to induce an effective immune response against microsatellite-unstable cancer. Many studies proved that microsatellite instability has a decisive role in the carcinogenesis and the malignant progression of head and neck cancer and, in the near future, it may become a useful tool in tailoring immunotherapy also in this field of precision oncology.

**Abstract:**

The mismatch repair (MMR) system has a major role in the detection and correction of DNA replication errors, resulting from DNA polymerase slippage or nucleotides misincorporation. Specific inherited/acquired alterations or epigenetic inactivation of MMR genes are associated with microsatellite instability (MSI): the loss of crucial function in repairing DNA alterations can promote carcinogenesis by favoring the accumulation of thousands of mutations in a broad spectrum of different anatomic sites such as colon, stomach, prostate, esophagus, endometrium, lung and head and neck. Recent extensive data suggest that tumor mutational burden strongly correlates with a clinical response to immunotherapy using checkpoint inhibitors and this response is influenced by MMR deficiency in a wide range of human solid cancers. In this context, few data about this crucial point are available for head and neck cancer (HNC). In this review, we discuss the role of MMR alterations and the resulting MSI in HNC pathogenesis. Furthermore, by summarizing the clinical available data on how they influence the progression of precancerous lesions and the risk of recurrence or second primary tumors, we want to define the current role of MSI in the management of HNC. Finally, we analyze the complex interaction between cancer cells and the immune system addressing the data now available about a potential correlation between microsatellite instability and immunotherapy response in HNC.

## 1. Introduction

Head and neck cancer (HNC) accounts for 4–6% of all human solid malignancies and about 50% of patients ultimately die of the disease, despite recent improvements in HNC diagnosis and treatment [1]. In the last decade, a better knowledge of the complex interactions between cancer cells and the host immune system has led to novel immunotherapy strategies that, also in the setting of recurrent/metastatic HNC, have shown encouraging results in comparison to conventional treatment [2]. The development of immune checkpoint inhibitors, targeting PD-1 (programmed cell death-1), such as nivolumab and pembrolizumab, or PD-L1 (programmed death-ligand 1), such as atezolizumab and durvalumab, has dramatically changed the therapeutic scenario for several types of cancer, including melanoma, lung, breast, kidney and bladder. In HNC patients, these new drugs have shown to yield objective response rates ranging from 17 to 36% and, because of their direct and indirect costs and toxicities, the identification of the subgroup of patients that is most likely to benefit is of the uttermost importance [2,3].

Among the many possible biomarkers predictive of an immune response in HNC patients, recent reports have suggested a potential role of microsatellite instability (MSI) in the setting of several human solid malignancies [4]. In this review, we discuss the role of MMR alterations and the resulting MSI in HNC pathogenesis. Furthermore, by summarizing the clinical available data on how they influence the progression of precancerous lesions and the risk of recurrence or of developing a second primary tumor, we want to define the current role of MSI in the management of HNC patients.

## 2. MMR System and Development of MSI: An Overview

**Figure 1:**
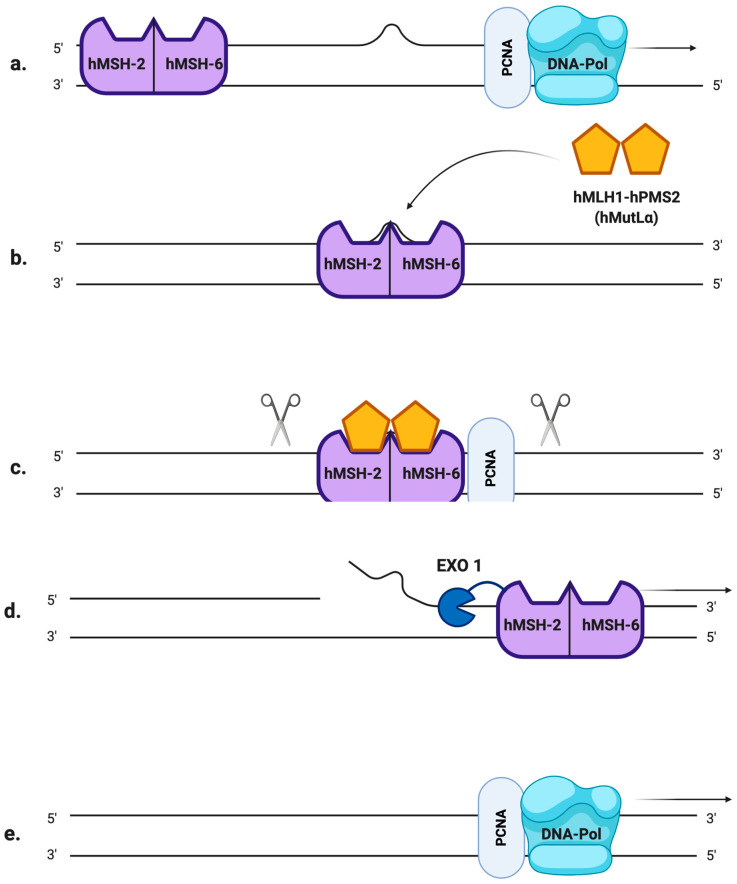
The mismatch repair (MMR) system (**a**) A mismatched base was wrongly incorporated in the newly synthesized DNA strand by DNA polymerase. (**b**) hMutSα detects the replication error and recruits hMutLα to form a heterodimer. (**c**) The newly synthesized strand is excised proximally and distally to the mismatched region by hMutLα, whose endonuclease activity is activated by PCNA. (**d**) hMutSα activates EXO1 that removes the excised region. (**e**) DNA polymerase carries out the DNA resynthesis.

Microsatellites, also referred to as simple sequence repeats (SSRs), are short tandem DNA sequences (usually 1–5 nucleotide long) that are repeated from 5 to 100 times, and that are thought to account for approximately 3% of the full human genetic code [5]. There are many existent microsatellite polymorphisms that differ from person to person, and they are all characterized by a high degree of heterozygosity: these peculiarities make them the ideal markers for genome mapping and the determination of the so-called “DNA fingerprint”, which has applications in many fields of medicine [6]. Usually, their lengths are very well conserved in the human genome but they are also particularly susceptible to inaccurate replication: the high rate of mutation in the number of repeats is mainly due to slippage phenomena of the DNA polymerase during the replication process [5]. If such an error occurs in healthy cells, it is promptly fixed by the mismatch repair system (MMR), a molecular machinery specialized in the detection and correction of replication mistakes, such as mispairing bases or small insertion/deletion loops (IDLs). In eukaryotic cells, the MMR system is composed of two different heterodimers: hMutS and hMutL [7]. The first one is the combination of the somatic protein hMSH2 (gene locus on 2p21) with either hMSH6 (2p16) or hMSH3 (5q14.1), resulting in hMutSα and hMutSβ heterodimers, respectively. On the other hand, hMutL is composed of hMLH1 (3p21) joined to hPMS2 (7q22.2), hPMS1 (2q31.1) or hMLH3 (14q24.3), resulting in hMutLα, hMutLβ and hMutLγ heterodimers, respectively [8]. The MSH2-MSH6 complex recognizes single base pair mismatches and 1–2 base IDLs (Figure 1a), while MSH2-MSH3 primarily recognizes larger IDLs. To remove the mispaired DNA sequence, MutSα (or MutSβ) recruits MutLα (Figure 1b), forming a tetrameric complex that proceeds to the excision step [9]: after PCNA (proliferating cell nuclear antigen, a component of the replication process) activates MutLα to incise the daughter strand far from the mismatched sequence (Figure 1c), its progressive excision is performed by the MutSα-activated exonuclease EXO1 (Figure 1d). Eventually, the DNA polymerases δ/ε carry out the DNA resynthesis [10] (Figure 1e).

The importance of the MMR in the replication process is proven by the fact that the error rate of DNA polymerases is estimated to be between 10^−4^ and 10^−5^ per nucleotide, whereas the activity of MMR can reduce the replication error-rate to 10^−9^–10^−10^ per nucleotide [11].

**Figure 2:**
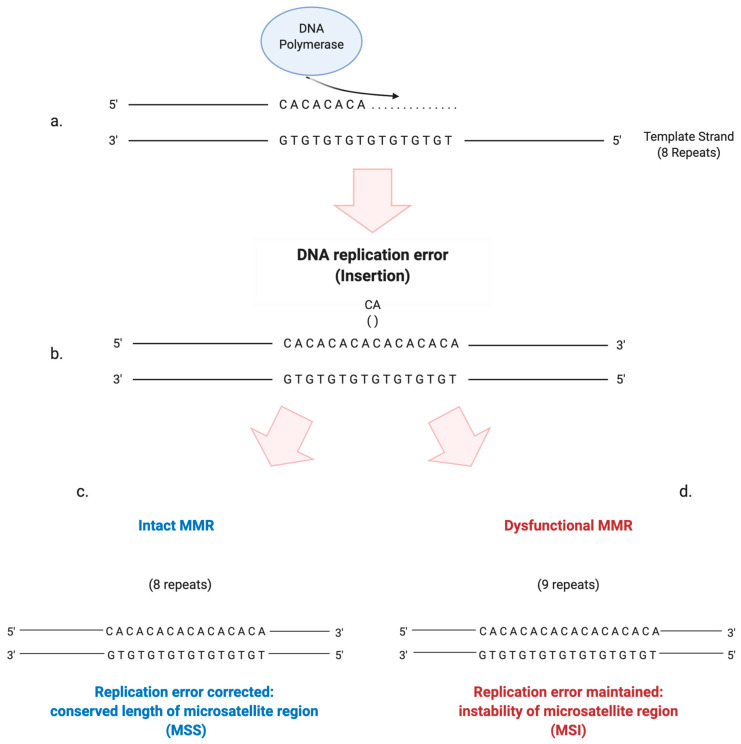
Schematic illustration of how the stability of microsatellite regions can be affected by the presence of a dysfunctional MMR system. (**a**) DNA polymerase performs the replication of a microsatellite sequence composed of 8 repeats of cytosine/adenine (CA). (**b**) Due to the slippage of DNA polymerase during replication, a CA repeat is wrongly incorporated. (**c**) If the MMR system is intact, the replication error is corrected and the right number of repeats is maintained. (**d**) If the MMR system is dysfunctional, the additional CA repeat will not be eliminated, leading to instability in the length of the microsatellite region.

An altered function of the MMR mechanism can lead to the development of a so-called “mutator phenotype”. It is characterized by the accumulation of frameshift and missense mutations in many loci, including coding and non-coding microsatellite sequences, that become highly unstable in their lengths [12] (Figure 2). Interestingly, it seems that the altered function or depletion of MutSβ is related to a mutator phenotype less than the depletion of MutSα: this suggests that large IDLs arise less frequently or that they can be more easily repaired [11]. The resultant MSI implies a distinctive carcinogenic pathway associated with altered expression of specific tumor suppressor genes and the activation of oncogenes that can eventually lead to malignant transformation [13]. The same frameshift mutations that lead to the onset of MSI, if occurring in coding sequences, can cause the creation of specific tumor “neoantigens”, aberrant proteins that can be recognized by the immune system as “non-self” [11].

In the context of DNA damage repair machinery, the correction of mispairing bases or small IDLs is the best known but not the exclusive function of MMR. Studies that focused on Fanconi anemia (FA)-associated mutations demonstrated that MMR proteins play an important role also in the regulation of the response to DNA interstrand crosslinks (ICLs) [14]. Biallelic germline alterations in one of the 22 genes implicated in the so-called “FA pathway” (like BRCA1, FANCD2, SLX4/FANCP and FANCJ) are the cause of this rare genetic disease, characterized by cellular hypersensitivity to DNA cross-linking agents, while its main clinical features are retarded growth, abnormalities of the skeletal system, bone marrow failure, development of acute myeloid leukemia and solid tumors (including HNC) at a young age [15]. FA-related genes are implicated in the multistep pathway to repair ICLs and the disease phenotype depends upon which one is mutated: for example, it seems that FA patients that tend to develop solid tumors are affected by a milder involvement of the bone marrow [15]. However, a recent review reported a 30% risk of cancer development in every FA patient, regardless of the specific mutation [16]. Among solid tumors, squamous cell carcinoma is by far the most frequent in these patients, with a 500–700-fold higher incidence compared to the general population and a typical tendency to head and neck localization [16]. Interestingly, many studies reported important interactions between MMR proteins and FA pathways components: the direct interaction between FANCJ helicase and MLH1, for example, seems to be essential for ICLs repair, and the lack of proficient coordination between the two leads to increased DNA cross-linking agents sensitivity, just like in FA cells [17]. MSH2 was shown as well to influence this molecular pathway because its depletion/inactivation results in the weakening of DNA damage response to ICLs, even if a dysfunctional FANCJ-MLH1 interaction is present: the resulting accumulation of an increasing number of aberrations promotes malignant transformation [18].

## 3. A Historical Perspective: MSI in Colorectal Cancer

MSI is historically considered to be the hallmark of one type of colorectal cancer (CRC) [19,20,21]. MSI was in fact discovered almost thirty years ago, when several research groups looking for molecular alterations or altered metabolic pathways that could be correlated to the development of CRC found that 10–15% of the sporadic cases were MSI+ [22,23]. Interestingly, they also observed that the presence of MSI could reach rates > 90% when testing patients affected by the Lynch syndrome (or HNPCC, hereditary non-polyposis colorectal carcinoma), the most common form of inherited cancer predisposition, and how this specific kind of genomic instability was related to the mutation or altered expression of DNA MMR-pathway proteins [6,24]. Since then, the identification of MSI patterns has become a fundamental step in the diagnostic process of both sporadic and hereditary CRC patients: MSI+ sporadic CRC is usually related to the methylation of the promoter region of MLH-1, is predominantly located in the proximal colon and is poorly differentiated [6,23]. Often the susceptibility to chemotherapy, in particular to 5-FU, is altered: a few trials in the past concluded that fluorouracil-based adjuvant regimes did not show significant efficacy in the treatment of patients affected by highly unstable CRCs [24,25,26]. However, the actual reliability of MSI as a predictive biomarker of chemotherapy efficacy in CRC patients remains uncertain, due to conflicting evidence [27,28]. On the other hand, MSI+ CRC seems to have a higher radiation sensitivity, which is related to general genome instability with a direct implication of functional MMR proteins in DNA damage response induced by radiation [29]. Many studies also highlighted how MSI+ CRC is associated with increased survival and in general better prognosis in comparison to their genetic stable counterparts: they are in fact characterized by a lower incidence of distant metastasis and dense infiltrates of lymphocytes among the neoplastic tissue, a fact that deposes for an intense immune response against the neoplasm [6].

These specific clinical, therapeutic and prognostic features also apply tor hereditary CRC: the assessment of MSI status and the research of the underlying mutations in MLH1, MSH2, MSH6 and PMS2 genes is now part of the diagnostic process of the Lynch syndrome, allowing early detection of CRC in asymptomatic patients who undergo an intensive surveillance program [6,21,23].

## 4. Laboratory Assessment of MSI

In 1998, the National Cancer Institute (NCI) proposed to test tumor and normal tissues using polymerase chain reaction (PCR) for five microsatellite markers as a standard procedure to detect MSI in CRC: two for mononucleotide repeats (BAT26 and BAT25) and three for dinucleotide repeats (D2S123, D5S346 and D17S250) [19]. The results of this analysis allow one to assess the grade of tumor MSI: MSI-high (MSI-H) for those with ≥30–40% marker instability (≥2 out of 5 markers); MSI-low (MSI-L) if marker instability is <30–40% (1 out of 5 markers); microsatellite stable (MSS) if no instability is detected [30,31]. PCR-based testing is considered the gold standard in the detection of MSI, but it is an expensive procedure and it always needs the control testing of non-malignant tissues [32]. The alternative method is immunohistochemical staining (IHC) for altered MMR proteins, a technique that is cheaper and more easily available than PCR [32]. IHC has, however, an important limitation: it can detect only quantitative alterations in protein expression, not qualitative ones [19,32]. For example, a missense mutation in the MLH1 gene sequence would not alter the entity of its expression, yet it would produce a protein incapable of combining with PMS2 to form a functional heterodimer. To avoid the possible misinterpretation of MSI status and increase the sensitivity of IHC is recommended to perform the immunostaining of all the four principal MMR proteins: PMS2 and MSH6, in fact, are degraded when incapable of forming a heterodimer with functionally altered MLH1 or MLH2. The depletion of the former would therefore prove the qualitative alteration of the latter [33]. PCR of microsatellite markers-consensus panels and MMR IHC staining are, at the present day, commonly accounted for as valuable screening tools, but some pitfalls have to be considered when interpreting the results of these analyses. It has been demonstrated, at least for CRC, that intralesional heterogeneity can lead to variable outcomes (notably, false-negative results) when determining tumoral MSI rates [34]. A precise microdissection of the samples is recommended in order to avoid analyzing stromal/inflammatory cells and to make sure to include at least 70% of tumor cells in the explored fragments [34]. Moreover, the detection of discordant patterns of MMR IHC staining may indicate the presence of distinct molecular aberrations in different tumor areas: in these cases, further investigation is strongly suggested in order to achieve a better therapeutic and prognostic orientation [35]. An experienced pathologist is therefore needed to perform a correct interpretation of the results of these molecular investigations.

## 5. MSI in Head and Neck Carcinogenesis

### 5.1. Impact on the Progression of Precancerous Lesions

In the last decades, many studies have been conducted to investigate the correlation between the development of MSI and HNC tumorigenesis [36]. While previous reviews [8,36,37] have dealt with this specific topic focusing mostly on oral squamous cell carcinoma (OSCC), we instead decided to extend our research to all types of HNC, including other anatomical subsites such as the larynx and salivary glands. Nonetheless, when MSI is concerned, OSCC remains the neoplastic lesion that has been more thoroughly studied. The results on the impact of MSI in HNC are summarized in Table 1.

Sengupta et al. [46] analyzed a group of patients affected by head and neck invasive or precancerous lesions: 50% of invasive squamous cell carcinoma (SCC) and 63% of dysplastic lesions showed promoter hypermethylation, a frequent epigenetic mechanism that inactivates gene transcription [68], of either or both hMLH1 and hMSH2. At least for the preneoplastic subset, the number of samples harboring MMR epigenetic inactivation was shown to be related to the grade of MSI detected, given that the hypermethylation of the MMR promoter grew proportionally with the degree of MSI. They also showed that tobacco use increased susceptibility to hMLH1 and hMSH2 hypermethylation not only in the affected lesion but also in the adjacent unaffected epithelium [46].

In 2011 Caldeira PC et al. [53,54] investigated this topic further and carried out two different studies demonstrating how hMLH1 expression decreased from low-grade oral leukoplakias to lesions showing severe dysplasia. Further studies highlighted the significant association of hMSH3 mutations with the development of leukoplakia and its progression to OSCC and that a decreasing trend in MMR expression can be detected when testing non-dysplastic samples and oral preneoplastic lesions with progressively increasing severity [61,65]. A lot of research was done on how the disruption of the MMR system is related to the development of OSCC: Ha PK et al. [40] described, in a cohort of 93 premalignant lesions of the upper aerodigestive tract and 18 invasive OSCC, a trend toward a higher MSI rate according to the histopathological severity of lesions: 6% in the hyperplastic lesions without atypia, 27% in the severe dysplasia/CIS and 33% in the invasive cancers.

The detection of MSI in OSCC samples seems to be significantly associated with the tumor stage, differentiation grade and the tendency to develop multiple oral malignancies [45,47,51].

As mentioned before, the decreased expression of MMR proteins, and hMLH1 in particular, can also be caused by promoter hypermethylation. In a matched case-control study, including 50 OSCC cases and 200 controls, promoter methylation of hMLH1 was detected (using methylation-specific PCR) in 38 (76%) of the cancers, but in none of the control samples. All the OSCC that harbored hMLH1 epigenetic alteration and tested negative for hMLH1 immunostaining were diagnosed at an early stage, thus reinforcing the concept that hMLH1 promoter hypermethylation seems to occur at the beginning of the OSCC carcinogenic pathway [56]. However, when considering the results of this particular study, it should be reminded that, although being a cheap and highly sensitive method for the DNA methylation quantification, methylation-specific PCR is burdened by a high rate of false-positive results [69].

Helal et al. [58] used immunohistochemistry to compare the pattern of expression of hMSH2 proteins between OSCC and oral dysplastic lesions (DL). Reduced expression of hMSH2 was detected in 26 of the 70 oral SCC (37.1%) and 2 of 21 oral DL (9.5%) with a statistically significant difference (*p* = 0.03), suggesting that the expression of the MMR system proteins diminishes as the histological severity of the lesion increases. These results were later confirmed by other authors, reinforcing the hypothesis of a direct correlation between MMR protein expression and dysplasia grade [64].

It seems that even inherited abnormalities of MMR protein genes can be associated with an increased risk or worse outcomes for HNC patients, particularly among smokers [63]. A study that genotyped 242 patients with tobacco-related OSCC and 205 healthy controls by polymerase chain reaction–restriction fragment length polymorphism (PCR–RFLP) technique, explored the risk related to the presence of the hMLH1-93 A > G (rs 1800734) single nucleotide polymorphism (SNP). It resulted that this specific polymorphism is associated with a higher risk of tobacco-related OSCC and could represent a useful screening marker [60]. The sample size of this study is quite low: even though a significant association of this specific SNP has been detected also for colorectal, lung and endometrial cancers [70,71,72], future larger studies (e.g., GWAS stratified by MSI status) are needed to corroborate these results.

**Figure 3:**
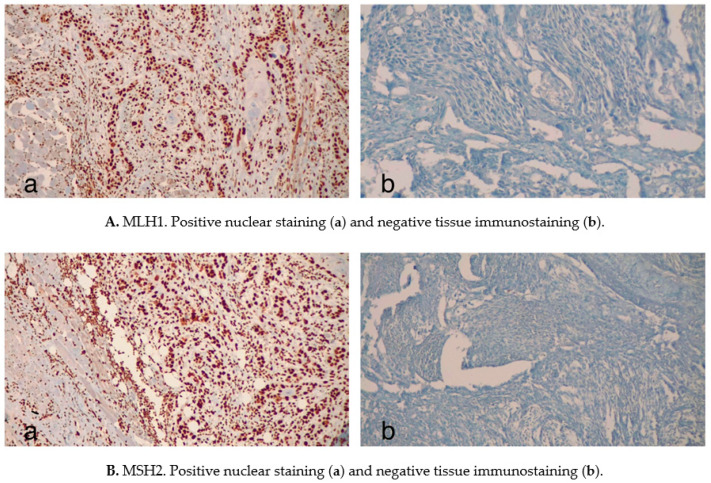
Some illustrative examples of MMR expression patterns in HNC specimens. Immunohistochemical staining (IHC), which is performed to determine the expression of MMR components such as MLH1 (**A**) or MSH2 (**B**), represents a useful tool to discriminate between MMR proficient/deficient tissues. All images are shown at 20× magnification. Images reproduced with permission from the archives of the Department of Pathology, Careggi University Hospital.

The malfunction of MMR and the reduced expression of its proteins not only correlate with the progression from preneoplastic lesions to OSCC but also with the stage and the behavior of oral invasive malignancies. High hMSH2 (Figure 3(Ba)) and hMLH1 expression (Figure 3(Aa)) have been associated with a reduced depth of invasion, and the absence of perineural invasion [57], while MMR deficient tumors (Figure 3(Ab,Bb)) have shown higher rates of bone invasion and high pT stage, and the presence of metachronous neoplasms [67].

Among the articles analyzed, only the paper by Pimenta et al. considered oral lichen planus (OLP): the actual risk for malignant transformation of OLP is controversial, as just some studies support its premalignant nature [73,74]. The actual overall frequency of OLP malignant transformation is estimated to be between 0.3 and 12.5% [73,74]. They examined the expression of hMSH2 protein by immunohistochemistry in 26 cases of OLP and in 10 samples of non-malignant mucosa as a control group. The percentage of cells expressing hMSH2 in reticular and atrophic/erosive subtypes of OLP was lower (46.54% and 48.79%, respectively) compared to normal mucosa (61.29%). It is possible that the reduced expression of the hMSH2 protein makes the epithelium of OLP more susceptible to DNA mutation, making it prone to OSCC development [42].

Some of the papers dealt with the process of carcinogenesis in the lips and its relation to MMR dysfunctions: Souza et al. [55] tested (in normal, dysplastic and malignant lip epithelium) the tissue expression of hMSH2 and other DNA repair proteins (p53, a most important tumor suppressor gene, APE1, a multifunctional enzyme of the base excision repair pathway, and ERCC1, a component of the nucleotide excision repair mechanism), finding a significant reduction in epithelial cell expression of these proteins. Other authors observed a decrease in the immunohistochemical determination of hMLH1 and hMSH2 in line with the increasing histologic grade. The largest number of MSI+ cells was detected in biopsies of actinic cheilitis without or with mild dysplasia, intermediate values were obtained for lesions with moderate/severe dysplasia and the lowest number of positive cells was presented by lower lip SCC [59,62,66].

Moving to other anatomical districts, Yalniz et al. [50] found that in their group of 99 patients (65 affected by laryngeal cancer) 26 were positive for MSI, with 17 patients displaying instability at more than one locus (MSI-H). In 2006 Demokan et al. [44] observed even higher rates. Among a cohort of 116 patients with many types of HNC (85 larynx, 13 salivary glands, 10 oral cavity, 6 nasal/paranasal sinus and 1 glomus), MSI was detected in the 41% of the samples and in the 59% of cases the promoter region of hMLH1 or hMSH2 resulted hypermethylated.

The development of MSI seems to be an indicator of a higher risk of malignant progression also in the larynx. Dysplastic laryngeal lesions from patients already affected by laryngeal cancer have significantly higher rates of MSI in comparison to preneoplastic lesions from otherwise healthy patients [75].

The presence of MSI and its relation with malignant evolution was also studied for the most frequent benign salivary gland tumor, pleomorphic adenoma (PA), with a malignant transformation rate around 3–4%. Despite the presence of a hyalinized stroma and focal calcifications are proven to be predictive for the risk of cancer development, a lot of different histologic features are only considered suggestive of such a risk, including cytologic atypia, increased mitoses and invasion of the capsule [76,77]. Researching for a useful immunohistochemical marker, Tobón-Arroyave et al. [48] conducted a study to examine the relationship between all those carcinogenesis-related histopathological features and the immunoexpression of hMLH1 and hMSH2 proteins in a group of 35 benign PA lesions, divided into low- and a high-risk subtype. Their findings were coherent with other papers that dealt with preneoplastic lesions of different head and neck districts: the mean expression values of both hMLH1 and hMSH2 were significantly lower in the high-risk subtype.

While the majority of authors agree in recognizing the significant prevalence of MSI and MMR dysfunctions in HNC and their role in neoplastic progression, few groups have obtained opposite results: De Schutter et al. in 2009 have, for example, concluded that MMR deficiency contributes little to the carcinogenic process of HNC, reporting that just one out of the eighty patients enrolled in their study was found positive for MSI [49].

Similar findings were obtained in two articles investigating MSI prevalence and hMSH2/hMLH1 altered expression in salivary gland tumors, either benign or malignant: a low-frequency microsatellite instability and no significant hMSH2/hMLH1 expression difference between benign and malignant neoplasms were detected [39,41].

A Canadian study was instead able to find a significant correlation between HNSCC and the presence of MSI, but not with MMR proteins dysfunction: investigating MSI status and hMLH1 and hMSH2 expression among 24 young HNSCC patients (≤44 years old; 46% smokers) and comparing it with an older cohort (33 HNSCC cases, ≥45 years, 88% with a history of tobacco abuse), they found that 100% of tumors in the younger cohort were MSI+ at least at one site, while 61% MSI+ tumors were found among the older patients [38]. The authors showed MSI to be more frequent in the non-smoker group and that it could be mainly detected in early-stage HNC, suggesting that genomic instability does occur in the early phases of tumor initiation/progression; however, they could not find any correlation between the inactivation of hMLH1 and hMSH2 genes and the development of MSI [38].

Of course, some of the included studies were limited by the small size of their cohorts of patients; furthermore, we have to consider that the anatomopathological methods to assess microsatellite instability and MMR protein expression are far from being standardized. The aforementioned methodological pitfalls of microsatellite markers-PCR and MMR IHC staining must not be underestimated; nonetheless, the consistency and the statistical significance of all these results cannot be ignored.

### 5.2. Impact on Local Recurrence

An interesting application of MSI-determination is the one performed on histopathologically tumor-free surgical margins [75,78]. Based on the assumption that the detection of instability in the microsatellite sequences length underlies a genotype able to trigger the malignant transformation, a few authors focused on the investigation of MSI markers on the negative margins they obtained after performing “radical” surgical resection of head and neck cancers, in order to see if it could represent a significant predictor of the risk of local recurrence.

As early as 2000, Sardi et al. used PCR to assess the presence of frame-shift alterations or loss of heterozygosity (LOH) of 10 different microsatellite markers in the surgical margins of a cohort of 41 patients affected by HNC. Among the 25 samples that resulted tumor-negative after histopathological analysis, 11 showed the same microsatellite alterations as in the primary tumors: seven of those patients experienced a local recurrence, while just one of the 14 tumors with MSI-negative margins recurred. Performing regression analysis the authors showed how molecularly positive margins were in fact an independent prognostic factor for local recurrence (*p* = 0.04) [75].

A study with a similar design tested five different tetranucleotide microsatellite markers on tumor samples and corresponding surgical margins from 54 patients who had undergone surgery for HNC and whose margins resulted in a microscopically negative status. Twenty-six (48%) tumors were positive for MSI and seven (27%) out of them had the same instability pattern in the paired margins. Of those seven patients, five developed a local relapse, suggesting an independent association between detection of MSI in the surgical margins and local recurrence of the tumor [78].

More recently, two other studies have tested a large cohort of OSCC cases using microsatellite markers on histopathologically negative surgical margins. The same conclusions were reached: the detection of microsatellite instability in tumor-free margins was related in a statistically significant manner to a higher risk of local recurrence [79,80].

All these studies ultimately suggest that molecular assessment of surgical margins’ microsatellite status could be a useful tool in HNC patients’ management to identify the subgroup that needs a more intense follow-up.

### 5.3. MSI as a Risk Factor for Multiple Primary Cancers

It is well known how several risk factors, such as tobacco and alcohol consumption or human papillomavirus (HPV) infection, have been associated with the development of multiple mutations and carcinogenic genotype in wide areas of tissue that can lead to disease progression, cancer relapse and multiple primary malignancies, according to a “field cancerization” model [81,82]. In addition, genetic factors may contribute to the development of a mutator phenotype, and molecular markers of genetic instability, such as MSI markers, could be a useful tool to identify patients particularly susceptible to the development of multiple primary cancers (MPC) in these districts [83].

A recent review [84] investigated this topic, focusing on various inherited and acquired gene mutations (germ-line mutations, single-nucleotide polymorphism, chromosomal instability, microsatellite instability and DNA methylation) in head and neck malignancies and researching the literature to find if each of them could have a correlation with increased susceptibility to MPCs. For MSI the authors considered just one study, performed in 1998 by Piccinin et al., in which MSI was analyzed at 20 chromosomal loci in 67 HNSCC patients (45 with single cancer and 22 with multiple primary tumors). The 22 MPCs cases were also searched for hMLH1 gene mutations, to evaluate its possible involvement in MSI development. They found neither a significant difference in the frequency of MSI comparing MPCs and single cancer cases nor somatic or germline mutations of the hMLH1 gene in microsatellite instable neoplasms [85].

Other studies reached different conclusions: in 2005 an Italian team enrolled 32 patients suffering from a new tumor on the “field” of a previously surgically treated oral or oropharyngeal carcinoma. They tested a panel of eight different microsatellite markers that were specifically selected to investigate the status of oncogenes/tumor suppressor genes such as p16 and p53. They retrieved samples from both the primary tumor and the newly developed one, finding that only eight of them showed an identical clonal pattern of microsatellite abnormalities, confirming that the more recent tumor was an actual recurrence of the index one. On the other hand, 15 patients (46.9%) showed different patterns of MSI in the two samples: the different genetic arrangement suggests an independent origin of the tumor, yet it supports the idea of a “field cancerization” related to genomic instability that induces the development of new genetic mutations and, as a consequence, second primary tumors [82,86].

More recently, other authors obtained results that seem to confirm this hypothesis: the detection of MSI in the index tumor has been found to have a statistically significant correlation to the risk of developing a second primary tumor in a large cohort of HNC patients [87].

Finally, epigenetic alterations of MMR genes, whose dysfunctions are well known to be related to genomic instability, has shown a positive correlation with the risk of MPC: Czerninski et al. demonstrated how, in their cohort of OSCC, the 100% of patients affected by multiple oral malignancies tested positive for hypermethylation of hMLH1 or hMSH2 promoters [47].

## 6. MSI and Immunotherapy of HNC

HNCs are frequently diagnosed when already at stage III/IV of the disease and the standard therapy consists of the association of surgery, radiation and/or chemotherapy. Nevertheless, the average failure rates remain considerable (60% locoregional and 30% distant recurrence rate) [88]. Platinum-based chemotherapy is the regimen most commonly used and the development of biological resistance to this type of drug is a well-known issue in this setting. A study performed on cisplatin-resistant vs. cisplatin-sensitive HNSCC cell lines reported that the former had a significantly decreased expression of MLH1 compared to the latter [89], confirming previous evidence suggesting that a dysfunctional MMR is associated with a worse response to this type of chemotherapy [90,91]. The specific underlying mechanism of the MMR-deficiency related chemoresistance is yet to be clarified: most likely, the dysfunctional MMR acts as a component in a complex association with other genetic alterations, such as defective p53-mediated DNA damage response pathway, cell-death controlling factors or other mutations that determine a diminished drug uptake in cancer cells [92].

Searching for new and effective HNC treatment options, an increasingly important role is being attributed to immunotherapy, that has recently revolutionized a large part of the oncology practice: the assumption on which it is based is that the human immune system has the innate ability to recognize cancer cells through specific “neoantigens” that are the results of the transcription of mutated DNA sequences generated during the process of malignant transformation [93]. This protective mechanism is usually evaded by tumors through the acquisition of immunoresistance and multiple checkpoints pathways, which normally help to maintain self-tolerance, and can be exploited by cancer cells to evade immune surveillance [94]. The programmed cell death protein 1 (PD-1) has emerged in the last years as one of the most important of these inhibitory pathways, due to its role in regulating the activity of T cells [95]. The first reports of cancer overexpressing this transmembrane glycoprotein and its ligand (PD-L1) date back to 2002 on a mice population: PD1-high tumor cells showed enhanced invasiveness and they were immune to cytotoxic T cells lysis, but both effects resulted reversible by the administration of anti-PD-L1 antibodies [96]. It has been observed how highly genetically unstable tumors, such as the ones that developed the aforementioned “mutator phenotype” (MSI and a high number of deletion/insertion frameshift mutations), are the most susceptible to this type of therapy [97].

Cancer genetic instability can be assessed by analyzing its tumor mutational burden (TMB), defined as the total number of mutations per coding area in a tumor genome [95,98]. A tumor with a high TMB will produce a great quantity of aberrant transcriptional products: some of those abnormal proteins will be presented on the cell surface and recognized as “neoantigens”, stimulating the T cells activation and the releasing of cytokines in the tumor microenvironment [99]. As previously mentioned, a direct consequence of this process seems to be an efficient immune activation and, among patients treated with antibodies targeting PD1-/PD-L1, a high TMB was associated with improved overall survival and it proved to be a good predictor of response to immunotherapy [98]. Recent studies also showed that the detection of a MSI-H status in tumors of various histology is usually associated with a high TMB [100] and that MMR-deficient tumors are characterized by significantly higher rates of genomic mutations compared to MMR-naive cases [101]. These results seem to be confirmed by the fact that MSI+ cancers are indeed very immunogenic and are usually highly infiltrated with activated CD8+ cytotoxic T and T helper 1 lymphocytes, actively producing IFN-γ [102]. Interestingly interferons (IFNs), and IFN-γ in particular, happen to be the principal up-regulator of the expression of multiple immune checkpoint inhibitors, including the PD-1/PD-L1 pathway [99,102]. Summing up, the most immunogenic tumors seem to be the ones with the strongest inhibition of the innate immune response [99].

In recent years, many studies demonstrated that MMR deficiency and MSI, similarly to TMB, could predict the tumor response to immunotherapies consisting of the administration of anti-PD-1 (pembrolizumab and nivolumab) and anti-PD-L1 (atezolizumab and durvalumab) antibodies. D.T. Le et al., in two subsequent studies, showed how immune checkpoint blockade with pembrolizumab induced an objective radiographic response in 40–53% patients with MMR-deficient cancers originated from various organs, in contrast to MMR-proficient malignancies, which did not benefit from the therapy [4,101]. It seems that, in particular, an high load of insertion/deletion-related frameshift mutations, typically associated with an MMR dysfunction, is highly predictive of a good anti-PD-1 immunotherapy response and that, for MSI+ tumors, the clinical benefit of immune checkpoint blockade directly correlates with the intensity of MSI (MSI-H, MSI-L or MSS) [103].

Although the efficacy of checkpoint blockade therapies and its correlation to genomic instability have been thoroughly studied predominantly for colorectal cancer, anti-PD-1 immunotherapies have also been approved as a second-line treatment for other types of advanced/metastatic malignancies [95].

The use of pembrolizumab and nivolumab, in particular, was approved in 2016 by the Food and Drug Administration (FDA) for the treatment of recurrent or metastatic (R/M) HNSCC associated with disease progression after the first-line treatment, which commonly consists in a combination of 5-FU, cisplatin/carboplatin and cetuximab (EXTREME regimen) [104,105]. More recently, pembrolizumab has been approved by the FDA as a first-line treatment itself in R/M HNSCC: in monotherapy (only in patients expressing high rates of PD-L1) or in combination with standard chemotherapeutic agents [3].

Many trials have been carried out to determine the effective efficacy of anti-PD-1 therapies in advanced HN cancers: the Phase Ib trial KEYNOTE-012, in which pembrolizumab was administered to 60 patients affected by R/M HNSCC, resulted in an overall response rate (ORR) of 18%, 25% in human papillomavirus (HPV)-positive and 14% in HPV-negative patients [106]. It seems that HPV viral gene products could increase the immunogenicity of the tumor, thus improving the efficacy of the immune checkpoint blockade [104]. All the patients enrolled in that trial had PD-L1-positive immunostaining in at least 1% of cancer, stromal or inflammatory cells that constituted the tumor microenvironment [104]. In order to better understand the possible role of PD-L1 expression as a predictive biomarker for immune checkpoint blockade therapy in HN cancers, the follow-up expansion cohort of the KEYNOTE-012 allowed enrollment regardless of the PD-L1 expression status [107]. Furthermore, the authors chose to compare two different scoring methods to evaluate the PD-L1 IHC assay: the tumor proportion score (TPS), which is the percentage of viable tumor cells showing partial or complete membrane staining at any intensity, and the combined positive score (CPS), the number of PD-L1 staining cells (tumor cells, lymphocytes and macrophages) divided by the total number of viable tumor cells, multiplied by 100 [108]. When sorted using TPS, no significant difference in terms of response to pembrolizumab was found between positive (≥1%) and negative (<1%) PD-L1 expression tumors. Conversely, when the whole tumor microenvironment was considered by CPS, a statistically significant increase in the response rate to immunotherapy was observed: CPS ≥ 1% patients had an ORR of 22%, in contrast with the 4% ORR of the negative ones (CPS < 1%) [107].

A recent clinical review collected the results of twelve studies in which PD-1/PD-L1 inhibitors were administered to a total of 1088 R/M HNSCC patients: 93% of them were tested for PD-L1 expression rate (with different scoring methods) and 67% for the HPV status. The meta-analysis of the outcomes seems to confirm that higher rates of PD-L1 expression enhance the overall response rate (ORR) of these patients: 18.9% for expressers versus 8.8% for non-expressers. On the other hand, in contrast to previous suggestions, no differences were observed in survival or tumor response among the HPV positive patients [109].

It is important to highlight that, even if the choice of CPS as a scoring method to evaluate the PD-L1 expression seems to strengthen its predictive potential [107], the results of the most recent trials where pembrolizumab was administered to R/M HNSCC patients seem to suggest that anti-PD-L1 immunotherapy should not be restricted only to PD-L1 expressing tumors. The authors of Phase II study KEYNOTE-055 [110] in fact, although observing higher response rates in patients with higher PD-L1 expression, reported a clinically meaningful response also in those that tested negative to immunostaining (18% ORR in CPS ≥ 1% vs. 12% ORR in CPS < 1% patients). Furthermore, 6- and 12-month progression-free survival and overall survival rates were similar in the two groups.

Similar conclusions were reached in the Phase III study KEYNOTE-048 [3]: 882 participants affected by untreated locally incurable R/M HNSCC, stratified by performance status, p16 status and PD-L1 CPS, were randomized in three groups and received, respectively, pembrolizumab alone, pembrolizumab with chemotherapy (platinum and 5-fluorouracil) and cetuximab with chemotherapy. The results demonstrated that pembrolizumab monotherapy significantly improved overall survival (OS) in PD-L1 CPS ≥ 1% patients while having the non-inferior OS, a longer duration of response and a better safety profile when compared to cetuximab with chemotherapy. Moreover, pembrolizumab with chemotherapy significantly improved OS, prolonged duration of response and had a similar safety profile versus cetuximab with chemotherapy in the whole population [3].

In conclusion, while PD-L1 CPS ≥ 1% appears to be a useful biomarker of favorable clinical outcomes of immunotherapy in HN cancers, the lack of its expression does not seem to be a consistent negative predictor of response to the immune checkpoint blockade. A recent case report seems to confirm this hypothesis but also opens up to new interesting possibilities: a 62-year-old man presenting an advanced SCC (T4N2M0) of the right piriform sinus that underwent standard induction chemotherapy followed by platinum-based radiochemotherapy, pharyngo-laryngectomy and unilateral cervical dissection for a first local recurrence and stereotactic reirradiation for a second one. A third loco-regional recurrence was found to be inaccessible neither by surgery nor radiotherapy. The patient was eventually enrolled in a Phase II trial and underwent the administration of anti-PD-L1. After five infusions, a complete clinical and radiological response was observed, despite the immunohistochemical PD-L1 expression status being proven to be negative. Interestingly, PCR and IHC performed on tumor samples demonstrated the presence of MSI-H, MLH1 and PMS2 expression loss. Those findings suggest that the same “mutator phenotype” associated with objective response to immune checkpoint blockade in CRC could be found in HN cancers as well and that MSI and MMR impairment could possibly be used as a predictor of immunotherapy outcomes [111].

## 7. Conclusions

It appears clear that strong biomarkers of immunotherapy response in HNC are yet to be found. At present, immunohistochemical PD-L1 expression often fails to correctly predict response to PD-1 pathway inhibitors due to its poor positive and negative predictive values.

There is a large amount of evidence supporting the role that MSI and MMR dysfunction play in precancerous lesions and their progression to HNC, and in cancer recurrence and in the development of multiple primary tumors. We believe that future studies need to determine if the detection of MSI and MMR protein alterations can, analogously to CRC, represent a useful tool in tailoring immunotherapy against HNC.

## Figures and Tables

**Table 1:** A summary of the most important studies on the role of microsatellite instability (MSI) and MMR dysfunction in the progression of head and neck cancer (HNC) precancerous lesions. ACC, adenoid cystic carcinoma; CIS, carcinoma in situ; SCC, squamous cell carcinoma.

Authors	Year	Lesion	Site	Methods	Main Results
Wang et al. [38]	2001	SCC	Oral cavity	Methylation-sensitive enzyme + PCR amplification + Immunohistochemistry (hMSH2, hMLH1)	No correlation between hMLH1/hMSH2 genes inactivation and MSI development
Ohki et al. [39]	2001	Pleomorphic adenomas, salivary carcinomas	Salivary glands	PCR amplification + Immunohistochemistry (hMSH2)	No significant expression difference between benign and malignant tumors
Ha K et al. [40]	2002	Dysplasia, CIS, SCC	Oral cavity	PCR amplification	Increasing amount of MSI rate as histologic grade increases in severity
Castrilli et al. [41]	2002	Pleomorphic adenomas, Warthin’s tumors, malignant tumors	Salivary glands	Immunohistochemistry (hMSH2, hMLH1)	Benign neoplasms expressed lower levels of both hMSH2 and hMLH1 proteins compared to malignant tumors
Pimenta FJ et al. [42]	2004	Lichen planus	Oral cavity	Immunohistochemistry (hMSH2)	Reduced expression of hMSH2 in oral lichen planus elevates the risk of OSCCs
Sardi I et al. [43]	2006	Preinvasive lesions, invasive cancers	Larynx	PCR amplification	MSI+ preinvasive lesions are at higher risk of evolving in invasive cancers
Demokan S et al. [44]	2006	SCC, adenoma, ACC, Warthin tumor, sarcoma	Larynx, Salivary glands, Oral cavity, nasal/paranasal sinus, and nasopharynx, glomus	Methylation-sensitive enzyme + PCR amplification	MSI significantly associated with HN cancers
Sanguansin S et al. [45]	2006	SCC	Oral cavity	PCR amplification	Polymorphism of hMSH2 can be used as a biomarker for prognosis and follow-up in OSCC treatment
Sengupta S et al. [46]	2007	Leukoplakia and SCC	Oral cavity and larynx	Methylation-sensitive enzyme + PCR amplification + Immunohistochemistry (hMSH2, hMLH1)	Promoter regions hypermethylation has a positive correlation with tobacco use
Czerninski R et al [47]	2009	SCC	Oral cavity	Immunohistochemistry (hMSH2, hMLH1) + PCR amplification	OSCC presented hypermethylation of hMSH2 and hMLH1 promoters
Tobón-Arroyave SI et al. [48]	2009	Pleomorphic adenoma	Minor salivary glands	Immunohistochemistry (hMSH2, hMLH1)	Lower hMSH2/ hMLH1 expression correlated with a higher risk for malignant transformation
De Schutter H et al. [49]	2009	SCC	Generic H&N tumors	PCR amplification	MSI has a low prevalence in HNSCC
Yalniz Z et al. [50]	2010	SCC + unspecified other types	Larynx + unspecified other sites	PCR amplification	More than 1 every 4 HN cancer is positive for MSI
Ashazila MJJ et al. [51]	2011	SCC	Oral cavity	PCR amplification	MSI status significantly correlated with tumor stage and differentiation grade
Tawfik HM et al. [52]	2011	SCC	Generic H&N tumors	Immunohistochemistry (hMLH1) + methylation-specific PCR	86.7% of the sample showed hMLH1 promoter hypermethylation
Caldeira PC et al. [53]	2011	Leukoplakia	Oral cavity	Immunohistochemistry (hMLH1)	Lower expression in severe dysplasia
Caldeira PC et al. [54]	2011	Leukoplakia	Oral cavity	Immunohistochemistry (hMLH1)	Lower expression in severe dysplasia
Souza LR et al. [55]	2011	Actinic cheilitis and LSCC	Lips	Immunohistochemistry (hMSH2)	Premalignant and malignant lip disease exhibit changes in the expression of hMSH2 proteins
González-Ramírez et al. [56]	2011	SCC	Oral cavity	Immunohistochemistry (hMLH1) + methylation-specific PCR	hMLH1 promoter methylation seems to be related to early stage OSCC
Theocharis S et al. [57]	2011	SCC	Mobile tongue	Immunohistochemistry (hMSH2, hMLH1)	Higher hMSH2 and MLH1 expression is significantly associated with a lower stage
Helal Tel A et al. [58]	2012	SCC	Oral cavity	Immunohistochemistry (hMSH2)	Lower expression in OSCC
Sarmento DJS et al. [59]	2013	Actinic cheilitis and LSCC	Lips	Immunohistochemistry (hMSH2, hMLH1)	Lower expression in OSCC
Jha R et al. [60]	2013	SCC	Oral cavity	PCR amplification (hMLH1)	Polymorphism in this gene may be related to OSCC predisposition
Mondal P et al. [61]	2013	Leukoplakia and SCC	Oral cavity	PCR amplification	Polymorphism in hMSH3 may be related to OSCC predisposition
de Oliveira DH et al. [62]	2014	Actinic cheilitis	Lower lip	Immunohistochemistry (hMLH1)	Lower expression in severe dysplasia
Nogueria GAS et al. [63]	2015	SCC	Oral cavity	PCR amplification	Polymorphism in the MMR system may be related to OSCC predisposition
Jessri M et al. [64]	2015	SCC	Oral cavity	Immunohistochemistry (hMSH2, hMSH6, hMLH1, PMS2)	Focal lack of hMSH6 indicates carcinoma in situ
Jessri M et al. [65]	2015	Leukoplakia and SCC	Oral cavity	Immunohistochemistry (hMSH2, hMSH6, hMLH1, PMS2)	Lower expression in severe dysplasia (except hMSH6)
Lopes ML et al. [66]	2016	Actinic cheilitis and LSCC	Lips	Immunohistochemistry (hMSH2)	hMSH2 mutation seems to be related to early events in the cancerization process
Vasan K et al. [67]	2019	SCC	Oral cavity	Immunohistochemistry (hMSH2, hMSH6, hMLH1, PMS2)	MMR proteins loss in OSCC is associated with more advanced primary tumors
